# Computational Power of Asynchronously Tuned Automata Enhancing the Unfolded Edge of Chaos

**DOI:** 10.3390/e23111376

**Published:** 2021-10-20

**Authors:** Yukio-Pegio Gunji, Daisuke Uragami

**Affiliations:** 1 Department of Intermedia, Art and Science, School of Fundamental Science and Technology, Waseda University, 3-4-1, Ohkubo, Shinjuku, Tokyo 169-8555, Japan; 2 Department of Mathematical Engineering, College of Industrial Technology, Nihon University, 1-2-1, Izumi-cho, Narashino 275-8575, Japan; uragami.daisuke@nihon-u.ac.jp

**Keywords:** cellular automata, dissipative structure, computational universality, edge of chaos, asynchronous updating

## Abstract

Asynchronously tuned elementary cellular automata (AT-ECA) are described with respect to the relationship between active and passive updating, and that spells out the relationship between synchronous and asynchronous updating. Mutual tuning between synchronous and asynchronous updating can be interpreted as the model for dissipative structure, and that can reveal the critical property in the phase transition from order to chaos. Since asynchronous tuning easily makes behavior at the edge of chaos, the property of AT-ECA is called the unfolded edge of chaos. The computational power of AT-ECA is evaluated by the quantitative measure of computational universality and efficiency. It shows that the computational efficiency of AT-ECA is much higher than that of synchronous ECA and asynchronous ECA.

## 1. Introduction

Research on complex systems frequently claims the system is far from equilibrium [1]. While dissipative structure proposed by Prigogine evokes perturbations from the outside [2], most studies on complex systems claim that a macroscopic system consists of microscopic elements interacting with each other and that the property of perturbations can be embedded in microscopic nonlinear interactions [3,4,5]. Especially, since chaotic dynamics was proposed, researchers have tendencies to consider that even randomness can be embedded as the properties within a system [6] and is recently utilized in encryption [7,8]. Those ideas are also taken in the studies of cellular automata (CA), and that leads to the classification of cellular automata because some CA have high nonlinearity and others have none [9,10,11].

The idea of which the property of an element of a system can be defined in the form of dynamics still accelerates the attempt to find out specific dynamics embedding not only the perturbation (chaos) but also the properties mixing the order and chaos. It entails that specific dynamics embedding the order and chaos can be regarded as dynamics containing both the inside of the system as order and the outside of the system as chaos. These dynamics touch on two important aspects. The one is the issue of the relation between microscopic and macroscopic perspectives, for instance, the statistical mechanics and thermodynamics [12], and the other one is the issue of the edge of chaos or criticality [13,14,15]. Both aspects are based on the issue of how one can understand two different concepts which look like elements of binary opposition on one hand but might be related to each other.

In the relationship between statistical mechanics and thermodynamics, if they are equivalent to each other, it is theoretically easy to deal with the system. Therefore, there was an endeavor to find out the condition under which statistical mechanics and thermodynamics are equivalent to each other. The isolated system without thermal exchange allows entropy maximization equivalent to free energy minimization. The closed system with thermal exchange allows entropy maximization equivalent to thermodynamic potential minimization [12]. Those conditions were found by an endeavor to relate the macroscopic perspective with the microscopic perspective. How about a system far from equilibrium? While the attempt to relate the micro with the macro failed, the non-local property is introduced to contrast the microscopic to the macroscopic perspectives [16,17]. A novel general way to understand a pair of microscopic and macroscopic perspectives is required.

In the relationship between the inside and the outside, two concepts, the inside and outside might be opposite with each other. However, if the relationship is embedded in the form of specific dynamics, one can find special dynamics featuring the order and chaos, that is found at the critical state in the phase transition. The critical state is called the edge of chaos [13,14]. It implies that any interaction between the inside and outside can be expressed as dynamics and that the inside-dominant dynamics show the order pattern and the outside-dominant dynamics show the chaotic pattern. Not only dynamics in differential equations but CA also shows the edge of chaos. If one recalls the situation of the system far from equilibrium, the attempt to relate the inside with the outside might be failed because the natural system is more perturbed from the outside [18]. As well as the relationship between the microscopic and macroscopic perspectives, a novel way to understand a pair of the inside and the outside is also needed.

CA that are more perturbed might be realized by using asynchronous updating. It is well known that behaviors of CA with asynchronous updating are totally different from those of CA with synchronous updating [19,20,21,22]. In addition, various devices to implement perturbed CA were proposed in the context of unconventional computing [23,24]. Notwithstanding those endeavors, there is little research to systematically consider perturbed CA including asynchronous updating. Although we previously proposed asynchronously tuned elementary cellular automata (AT-ECA) and argue that most of them show critical properties independent of the transition rule [25,26,27], the construction of the updating is so complicated that the significance of it was unclear. In this paper, we define the active and passive updating in a term of the order of updating and define AT-ECA equipped with the device tuning the synchronous with the asynchronous updating. If asynchronous tuning is applied to ECA, then that leads to critical behavior which looks like behavior at the edge of chaos. The computability of AT-ECA is here estimated by a quantitative measure of computational universality and computational efficiency.

## 2. Asynchronously Tuned ECA (AT-ECA)

We previously proposed asynchronously tuned elementary cellular automata (AT-ECA) and showed their basic properties [25,26,27]. Since AT-ECA is proposed, compared to synchronous ECA that is usually used, we here first argue the significance of synchronous time.

Elementary cellular automata (ECA) are defined by a set of the binary sequences of cells, **B***^n^* with **B** = {0, 1} and a transition rule *f_r_*:**B**^3^→**B**, where *f_r_* is synchronously updated to all cells and *r* represents the rule number, where *r* = Σ^7^*_s_*_ = 0_2*^s^d_s_* with *d_s_* = *f_r_* (*x, y, z*) and *s* = 4*x* + 2*y* + *z*. The transition rule with synchronous updating is expressed as *a_i_^t^*^+1^ = *f_r_*(*a_i_*_-1_*^t^*, *a_i_^t^*, *a_i+_*_1_*^t^*). Given a binary sequence, a transition rule is globally and synchronously adapted to all cells [9,11]. Although synchronous updating is usually ignored, synchronous updating is a special assumption in biological systems. Imagine a multicellular system. Consider the updating of a cell, autonomously activated by the active updating, and the updating triggered by neighboring cells by the passive updating. If there is a temporal gap in the updating, one can find the difference between the active and passive updating with respect to the order of updating. The preceding cell’s updating is called active, and the following cell’s updating is called passive. Synchronous time invalidates the difference between active and passive updating because it looks as if a cell autonomously updates its state and simultaneously as if a cell updates its state triggered by neighboring cells. One cannot determine whether either of the active and passive updating is true in synchronous updating.

**Figure 1:**
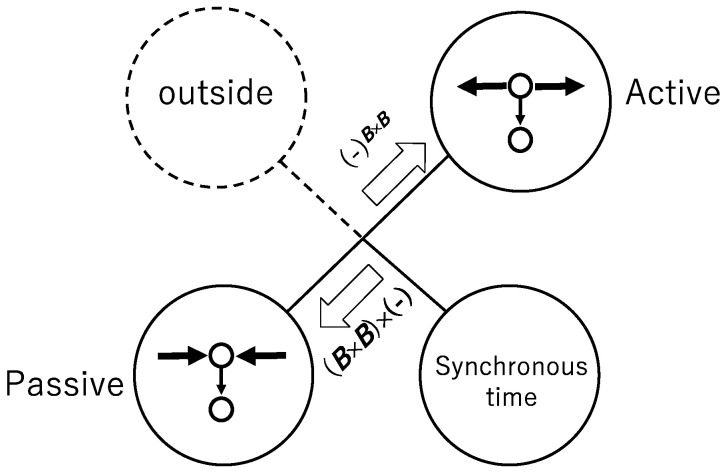
Schematic diagram for synchronous cellular automata. The isomorphism between the active and passive is based on the hidden assumption of synchronous time that plays a role as an interface.

Figure 1 shows the indistinguishability between the active and the passive by the straight line connecting the active circle and the passive circle and shows the hidden assumption founding the indistinguishability as synchronous time. We call the element on the left the interface. In ECA the synchronous time plays a role as the interface. Any perturbation outside ECA does not influence ECA, and that is shown by the outside surrounded by a broken circle. While one can couple the effect of perturbation with ECA, that perturbation is not “inevitable”. We here introduce the perturbation in a broad sense as the “inevitable”.

**Figure 2:**
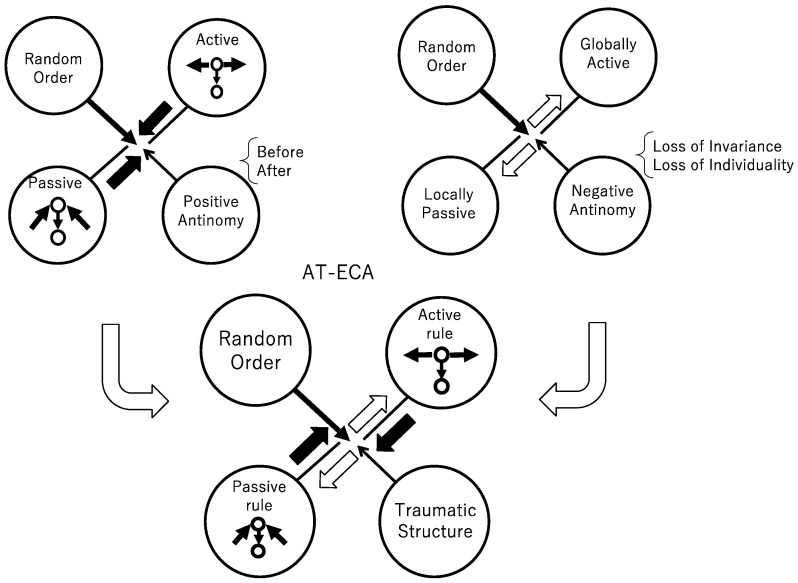
Schematic diagram for asynchronously tuned elementary cellular automata (AT-ECA). Coupling the positive with negative antinomy results in AT-ECA, where the coupling structure is called traumatic structure.

To implement the inevitable perturbation, we define the positive and negative antinomy, coupled them, and that leads to the construction of AT-ECA. Through this argument, one can find that AT-ECA is the inevitable dissipative structure. To implement inevitable perturbation, we first define the positive antinomy instead of the interface that is shown in Figure 1. The positive antinomy of the active and passive implies that each cell allows both active and passive updating. If the order of updating is given, and if a cell updates its state before its neighboring cells, then a cell is interpreted as active. Otherwise, a cell is interpreted as passive. Thus, if the order of updating is randomly given at each time, a cell can be active at some time step and can be passive at other time steps, depending on the order at that time step. That implements the positive antinomy of the active and passive antinomy, since the positive antinomy allows both active and passive updating that tends to connect them with each other, as shown in the diagram of Figure 2 (left) above. Next, we define the negative antinomy of the active and negative updating. With respect to a transition rule, one can find neither active and passive updating in a multicellular system. The active attitude to a transition rule can be defined as choosing one invariant transition rule. In contrast, the passive attitude can be defined as a changeable rule dependent on the order. Thus, if one defines both a passive rule and negative rule for each cell, and if the passive rule is invariant and the active rule is changeable, one can establish that neither active nor negative rule is accepted for each cell, and that implies that the notion of active and passive is separated from each other in the form of the negative antinomy, as shown in the diagram of Figure 2 (right) above. Coupling the two antinomies leads to the traumatic structure. Although trauma is a technical term in psychiatry, we here use the traumatic structure in a broader sense. Since the trauma implies an entangled state consisting of two antagonistic senses such as the sense of victim and of perpetrator, we call the entangled state of the positive and negative antinomy of the active and passive the traumatic structure (Figure 2 below). Since that an entangled state does not usually appear without a psychiatric disorder, that is immediately relevant for traumatic structure.

Asynchronously tuned ECA (AT-ECA) is defined by ECA equipped with the traumatic structure. To implement the positive antinomy of the active and passive updating, the order of updating for all cells, i ϵ {1,2,⋯, N} is defined by

(1) $$Ord^{t}\left(i\right)\;\epsilon \;\left\{1,2,\cdots ,\;N\right\}\;,\;i\neq j\Rightarrow Ord^{t}\left(i\right)\neq Ord^{t}\left(j\right).$$

A function,$\;Ord^{t}\left(i\right)\;$is defined as a bijective map, and implies an order of updating at the t-th step for the i-th cell. N implies a system size. In the case of N=5, $Ord^{t}\left(i\right)$ is expressed as

(2) $$\;Ord^{t}\left(0\right)=3,\;Ord^{t}\left(1\right)=5,\;Ord^{t}\left(2\right)=4,\;Ord^{t}\left(3\right)=1,\;Ord^{t}\left(4\right)=2.\;$$

In time development, $Ord^{t}\left(i\right)$ is randomly determined at each time step. By using $Ord^{t}\left(i\right)$, the state of each cell is updated.

As mentioned above, the traumatic structure is implemented by the active and passive rules for each cell. The passive rule that is invariant through time and universal for all cells is defined by

(3) $$\frac{000}{a_{0}},\;\frac{001}{a_{1}},\;\frac{010}{a_{2}},\;\frac{011}{a_{3}},\;\frac{100}{a_{4}},\;\frac{101}{a_{5}},\;\frac{110}{a_{6}},\;\frac{111}{a_{7}}\;,$$

where $a_{0}\sim a_{7}$ is either 0 or 1. In contrast, the active rule is defined such that it is different for each cell and is temporally changed. Thus, the active rule at the t-th step for the i-th cell is defined by

(4) $$\frac{000}{e_{i,\;0}^{t}},\;\frac{001}{e_{i,\;1}^{t}},\;\frac{010}{e_{i,2\;}^{t}},\;\frac{011}{e_{i,\;3}^{t}},\;\frac{100}{e_{i,\;4}^{t}},\;\frac{101}{e_{i,\;5}^{t}},\;\frac{110}{e_{i,\;6}^{t}},\;\frac{111}{e_{i,\;7}^{t}}\;$$

$e_{i,\;0}^{t}\;\sim \;e_{i,\;7}^{t}\;$is also either 0 or 1. It is assumed that for each cell the active rule is initially the same as the passive rule. It implies that for t=0, and for any i,

(5) $$\;e_{i,\;k}^{0}=a_{k}\;\left(k=0,\cdots ,7\right)\;.\;$$

In t>0, $e_{i,\;k}^{t}$ is determined at each step by the procedure mentioned later.

The state of the i-th cell at the t –th step expressed as $c_{i}\left(t\right)$ is defined as

(6) $$c_{i}\left(t\right)\in \left\{0,1\right\}\;.\;$$

Initially $c_{i}\left(0\right)$ is randomly distributed in an array.

The state of each cell is updated, depending on the local order of updating that is the order of $Ord^{t}\left(i-1\right)$, $Ord^{t}\left(i\right)$, and $Ord^{t}\left(i+1\right)$. The condition of that updating is divided into the following four cases.

(7) $$\mathrm{If}\;Ord^{t}\left(i-1\right)>Ord^{t}\left(i\right)<Ord^{t}\left(i+1\right)\;,\;\;c_{i}\left(t+1\right)=g_{i}^{t}\left(c_{i-1}\left(t\right),\;c_{i}\left(t\right),c_{i+1}\left(t\right)\right);$$

(8) $$\mathrm{If}\;Ord^{t}\left(i-1\right)<Ord^{t}\left(i\right)<Ord^{t}\left(i+1\right)\;,\;c_{i}\left(t+1\right)=f\left(c_{i-1}\left(t+1\right),\;c_{i}\left(t\right),c_{i+1}\left(t\right)\right);$$

(9) $$\mathrm{If}\;Ord^{t}\left(i-1\right)>Ord^{t}\left(i\right)>Ord^{t}\left(i+1\right),\;c_{i}\left(t+1\right)=f\left(c_{i-1}\left(t\right),\;c_{i}\left(t\right),c_{i+1}\left(t+1\right)\right);$$

(10) $$\mathrm{If}\;Ord^{t}\left(i-1\right)<Ord^{t}\left(i\right)>Ord^{t}\left(i+1\right),\;c_{i}\left(t+1\right)=f\left(c_{i-1}\left(t+1\right),\;c_{i}\left(t\right),c_{i+1}\left(t+1\right)\right)$$

where $c_{i}\left(t\right)$ represents the state of the i-th cell at the t-th step, and function, f and$\;g_{i}^{t}\;$represent the passive rule defined by Equation (3) and the active rule defined by Equation (4). Namely, for the passive rule, $f\left(0,\;0,\;0\right)=a_{0}$, $f\left(0,\;0,\;1\right)=a_{1}$, $f\left(0,\;1,\;0\right)=a_{2}$, $f\left(0,\;1,\;1\right)=a_{3}$, $f\left(1,\;0,\;0\right)=a_{4}$, $f\left(1,\;0,\;1\right)=a_{5}$, $f\left(1,\;1,\;0\right)=a_{6}$, $f\left(1,\;1,\;1\right)=a_{7}$. For the active rule, $g_{i}^{t}\left(0,\;0,\;0\right)=e_{i,\;0}^{t}$, $g_{i}^{t}\left(0,\;0,\;1\right)=e_{i,\;1}^{t}$, $g_{i}^{t}\left(0,\;1,\;0\right)=e_{i,2}^{t}$, $g_{i}^{t}\left(0,\;1,\;1\right)=e_{i,\;3}^{t}$, $g_{i}^{t}\left(1,\;0,\;0\right)=e_{i,\;4}^{t}$, $g_{i}^{t}\left(1,\;0,\;1\right)=e_{i,\;5}^{t}$, $g_{i}^{t}\left(1,\;1,\;0\right)=e_{i,6}^{t}$, $g_{i}^{t}\left(1,\;1,\;1\right)=e_{i,\;7}^{t}$.

Finally, the active rule is updated by the following procedure. The condition of updating the active rule is divided into three cases, depending on the updating of the state of the cell. For the first case, if the state of the cell is updated by following Equation (7), the active rule is not updated, and that implies

(11) $$e_{i,\;k}^{t+1}\;=\;e_{i,\;k}^{t}\;\left(k=0,\cdots ,7\right).\;$$

For the second case, if the state of the cell is updated by Equations (8) and (9), the active rule is updated by

(12) $$e_{i,\;k}^{t+1}=\{\begin{array}{c} a_{0}\;\left(k=m\right)\\ \;e_{i,\;k}^{t}\;\left(k\neq m\right) \end{array}\;,$$

where $m=4\cdot c_{i-1}\left(t\right)+2\cdot c_{i}\left(t\right)+c_{i+1}\left(t\right)\;$. It implies that the value corresponding to the neighborhood at the t-th step is replaced by $a_{0}$ that is defined in the passive rule. Equation (12) plays a role in initializing the active rule. For the third case, if the state of the cell is updated by Equation (10), the active rule is updated by

(13) $$e_{i,\;k}^{t+1}=\{\begin{array}{c} \;c_{i}\left(t+1\right)\;\left(k=m\right)\\ e_{i,\;k}^{t}\;\left(k\neq m\right) \end{array}.\;$$

**Figure 3:**
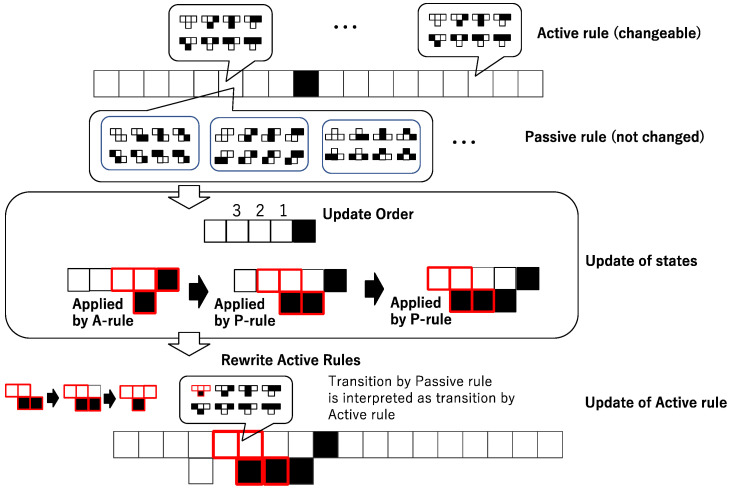
Schematic diagram of the adjustment of the passive and active rules. The above layer shows that each cell has both active and passive rules. The middle layer shows that the transition is based on the update order. The first active rule (A rule) is applied to the cell, and the passive rule (P rule) is applied. The bottom layer shows how the results of the application of the passive rule are interpreted as the application of the active rule, and that leads to the update of the active rule.

While each cell has both the passive and the active rules, they are tuned with each other in the form of Equation (13). Since Equation (13) invalidates the difference between the passive and active rules, the positive active/passive antinomy which contains both active and passive updating is weakened, and that also implies the negative active/passive antinomy. That tuning between the passive and the active rules is illustrated as shown in Figure 3. In Figure 3, any cell has the same active rule whose rule number is rule 22. Since the configuration of squares (cells) in vertical direction represents time, it is easy to see that the configuration of the active rule in Figure 3 represents the local order in the neighborhood such as $Ord^{t}\left(i-1\right)>Ord^{t}\left(i\right)<Ord^{t}\left(i+1\right)$. The passive rule of the sixth cell in Figure 3 is also rule 22, and is illustrated for three cases, $\;Ord^{t}\left(i-1\right)>Ord^{t}\left(i\right)>Ord^{t}\left(i+1\right)$, $Ord^{t}\left(i-1\right)<Ord^{t}\left(i\right)<Ord^{t}\left(i+1\right)$, and $\;Ord^{t}\left(i-1\right)<Ord^{t}\left(i\right)>Ord^{t}\left(i+1\right)$. For the sixth cell in Figure 3, $c_{i}\left(t+1\right)\;$= 1 and $c_{i-1}\left(t\right)=0,\;c_{i}\left(t\right)=0,\;c_{i+1}\left(t\right)=0$, and then the active rule is updated by the following: $e_{i,\;0}^{t+1}=c_{i}\left(t+1\right)=1\;$(0 = $4\cdot c_{i-1}\left(t\right)+2\cdot c_{i}\left(t\right)+c_{i+1}\left(t\right)\;$). The result of the tuning is drawn by red squares in the rewrite active rule in Figure 3.

**Figure 4:**
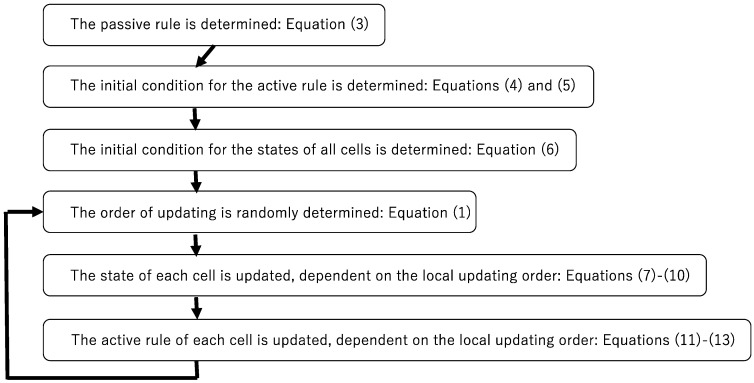
Algorithm for the asynchronously tuned automata.

Figure 4 shows the procedure of simulating asynchronously tuned automata. Although it looks so complicated compared to synchronous cellular automata, it reveals just perpetual adjustment between the passive and active rules.

**Figure 5:**
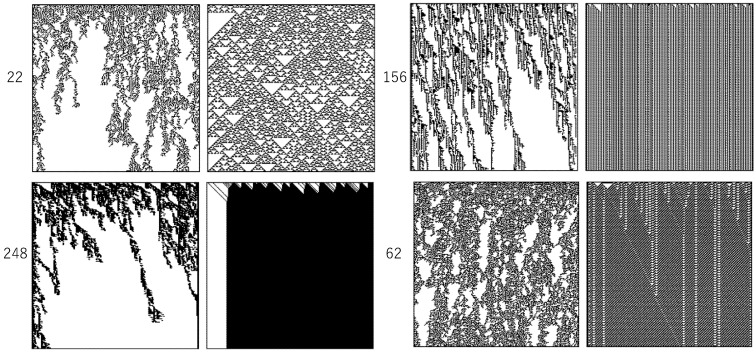
Pattern generated by AT-ECA (**left**) and synchronous ECA (**right**). The number represents the rule number of the passive rule of AT-ECA and the rule number of synchronous ECA.

Figure 5 shows some examples of patterns generated by asynchronously tuned cellular automata (AT-ECA). Each pair of squares shows a pattern generated by AT-ECA (left) and a pattern generated by synchronous ECA (right), where the passive rule of AT-ECA is the same as the rule of synchronous ECA, which is represented by the accompanying number. Due to the common rule number, one can say that the procedure of asynchronous tuning is applied to ECA. For the top left pair in Figure 5, the procedure of asynchronous tuning is applied to rule 22 of ECA. It is easy to see that asynchronous tuning lets the ECA exhibit class 4 like behavior, whether the original synchronous ECA are class 1, 2, or 3. Due to asynchronous tuning, the class 3 chaotic pattern generated by rule 22 shows a cluster-like pattern, and the class 2 local periodic pattern generated by rule 156 or 62 also shows a cluster-like pattern. Even if the original synchronous ECA shows the class 1 homogeneous pattern, asynchronous tuning changes the behavior to the cluster-like pattern.

We previously examined the behavior of all AT-ECA, 256 rules [25,26]. It is found that the behaviors of many ECA are changed from class 1, 2, and 3 to class 4. Although there is no order parameter controlling the phase from class 1, 2, 3, and 4, all ECA rules can be arranged in an ECA rule space, with respect to whether chaotic behavior appears or not. It leads to the phase transition from the order pattern (class 1 or 2) to the chaotic pattern (class 3) via the complex class 4 pattern. The phase transition from the order to chaos is similar to the phase transition from the solid phase (order) to the liquid (chaos) phase in H_2_O. Both transitions contain the critical phenomenon showing the intermediate state of order and chaos. Even in ECA, one can find class 4 behavior in rule 110, showing the intermediate complex pattern consisting of chaos and order. Rule 110 shows a complex periodic pattern that is locally oscillated and sometimes fires spatially propagated solitary waves (called gliders). If those patterns are disturbed by perturbation, patterns show typical cluster-like patterns, of which locally stable patterns containing spatially propagating elements. Although it is not known that rule 110 shows an extrinsic critical character such as power law distribution, it is considered that rule 110 is located as the critical phenomenon in the phase transition from order to chaos. The critical state in the transition from order to chaos is sometimes called the edge of chaos.

In terms of computability, one can see computation in patterns generated by ECA. If configurations of cells whose values are 1 are compared to the values in computation, it is considered that class 1 or 2 ECA shows a highly efficient computation since a specific stable pattern is rapidly obtained from random initial conditions. In contrast, it is considered that class 3 ECA shows a universal computation since various configurations can be obtained from the initial conditions. While an important role in computation is efficient and universal computation, which can be realized neither by class 1 and 2 nor by class 3 ECA. It is known that rule 110 showing class 4 can be used as a universal Turing machine. Since locally periodic patterns interact with each other via the gliders, rule 110 can show both universal and highly efficient computation to some extent. That computability might be taken after by cellular automata showing cluster-like patterns.

**Figure 6:**
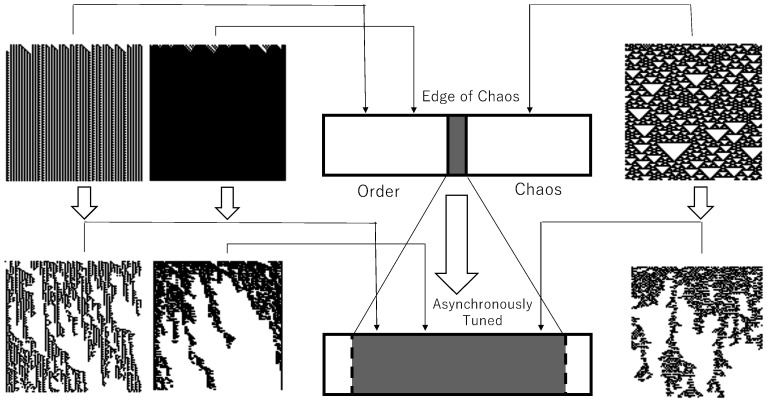
Unfolded edge of chaos by asynchronous tuning.

Those considerations can lead to schematic diagrams showing the unfolded edge of chaos, as shown in Figure 6. A diagram in the above center shows a schematic diagram of the edge of chaos found in synchronous ECA rule space. It is assumed that all 256 ECA rules are arranged depending on whether their generated patterns are chaos or not. In ECA rules located at the edge of chaos there are only four rules which are symmetric to rule 110 with respect to local configurations or values. Typical patterns generated by synchronous ECA are shown, such that class 2 and class 1 are classified by order pattern, and class 3 are classified by chaos. Those synchronous ECA patterns are shown in Figure 6 above and connected to the phase transition by thin arrows, while thick arrows represent the application of asynchronous tuning to synchronous ECA. As well, in Figure 5, both order and chaos can be replaced by cluster-like patterns corresponding to the edge of chaos. Two patterns connected by thick arrows have the same rule of ECA as well as the case of Figure 5, and i.e., if synchronous ECA is rule *r*, the passive rule of AT-ECA is also rule *r*. Since the application of asynchronous tuning to synchronous ECA changes generated patterns from both chaos and order to the cluster-like patterns at the edge of chaos for many cases, one could say that the edge of chaos is unfolded by asynchronous tuning, as shown in Figure 6.

Not only patterns, but the power law distribution of the temporal decay of the density and of the power spectrum is estimated for AT-ECA. It is found that the pattern generated by AT-ECA shows the power law distribution of the decay of the density of state-1, and that the exponent of the power law is close to the exponent found at the critical point in the phase transition of the directed percolation. It is also found that the time series of decimal expression for the binary sequence generated by AT-ECA shows, what is called, 1/*f* noise. Those results directly support that AT-ECA shows critical behavior or behaviors found at the edge of chaos. In that sense, one can justify the idea of unfolding edge of chaos of which the critical phenomenon found only at the narrow area of the critical point in synchronous ECA can be unfolded in the rule space of ECA and the critical behaviors are ubiquitously found in the rule space.

The idea of the “unfolding edge of chaos” could explore the idea of criticality. Not only ECA but dynamic systems show the edge of chaos in the phase transition in which there is a very narrow region showing criticality, and that is generalized in the world of natural systems. Since most biological systems are interpreted as systems adapted to their own environments to some extent, they are regarded as systems at the edge of chaos. The next question arises, how do rare systems at the critical point become ubiquitously found in biological systems. Most researchers think that the solution is natural selection, and that tuning toward the criticality is driven by natural selection. That idea leads to the idea of self-organized criticality. Although self-organized criticality could explain the reason why the critical state is ubiquitously found in the natural world, the fitness under the environments is required to define self-organized criticality, and that evokes the role of natural selection too much. The idea of unfolding the edge of chaos implies that critical phenomena are ubiquitously found without severe selection.

To understand the role of the unfolding edge of chaos, it is necessary to examine the computability of AT-ECA. We propose the measure of universality and efficiency of computability of ECA and compare the measurements obtained by synchronous ECA with that by asynchronous ECA. We here examine that measure obtained by AT-ECA.

## 3. Computability of AT-ECA

The computability of AT-ECA is measured with respect to the following computational universality and computational efficiency [28]. First, computational universality is defined by the following. Given 2*^n^* all possible initial states with random boundary conditions, the computational universality of rule *r*, *U*(*r*), is defined by

(14) $$\;S_{R}\left(r\right)=\{\;G\left(f_{r}^{T}\right)\left(c_{0},\;c_{1},\;\ldots ,\;c_{n+1}\right)\in B^{n}|\;\left(c_{1},\;\ldots ,\;c_{n}\right)\in \;B^{n},\;\left(c_{0},\;c_{n+1}\right)\in R\left(B^{2}\right)\}\;$$

(15) $$U\left(r\right)=\#S_{R}\left(r\right)$$

(16) $$U_{N}\left(r\right)=\frac{U\left(r\right)}{2^{n}}$$

where for a set S, #S represents the cardinality of a set S, $R\left(B^{2}\right)$ represents one element set randomly determined from $B^{2}$, superscript T represents T numbers iteration of a transition rule $f_{r}$. If n=3, then U(0)=#{(0, 0, 0)}=1, and U(204)=#{(0, 0, 0), (0, 0, 1), (0, 1, 0), (0, 1, 1), (1, 0, 0), (1, 0, 1), (1, 1, 0), (1, 1, 1)}=8. $U_{N}\left(r\right)$ represents the normalized computational universality. Here, we call elements of a set, $S_{R}\left(r\right)$, reachable states or possible goals.

Next, we define the computational efficiency of a transition rule r. To separate from the computational universality, the computational efficiency is expressed by the average time to reach the reachable states. For each reachable state $X\in S_{R}\left(r\right)$, the average time to reach *X* is represented by $\tau_{r}\left(X\right)$ is expressed as

(17) $$\tau_{r}\left(X\right)=\sum_{Y\in B^{*}}T(G(f_{r}^{T})\left(Y\right)=X)$$

where $B*\;=B^{n}\times R\left(B^{2}\right)$, $T\left(G\left(f_{r}^{T}\right)\left(Y\right)=X\right)$ implies time T such that $G\left(f_{r}^{T}\right)\left(Y\right)=X$. Additionally, if$\;G\left(f_{r}^{T}\right)\left(Y\right)=X$ is not obtained within $2^{n}$ time steps, then $T\left(G\left(f_{r}^{T}\right)\left(Y\right)=X\right)$ is a constant value, $T_{\theta }$. For the case of R204 in which any initial condition is not changed by the transition, $G\left(f_{r}\right)\left(Y\right)=Y$ with T=1 and then for any $X\in S_{R}\left(r\right)$, $\tau_{r}\left(X\right)=\left(1+T_{\theta }\left(\#B*\;-1\right)\right)$, since any initial condition except for X cannot reach the goal X. The computational efficiency is defined by

(18) $$E\left(r\right)=\sum_{X\in S_{R}\left(r\right)}\begin{array}{c} \frac{\tau_{r}\left(X\right)}{\#S_{R}\left(r\right)} \end{array}$$

Since E(r) is the average time to reach the reachable state, the smaller E(r) is, the more efficient ECA r is. By using that computational universality and computational efficiency, we here examine the computability of AT-ECA.

It is easy to imagine that computational universality trades off with computational efficiency. Conrad previously claimed that molecular computing shows the tradeoff between computational universality and efficiency. We previously visualized the tradeoff between computational universality and efficiency in synchronous ECA, the step function outputting the discretized computational efficiency for the semi-interval in the interval [1,255] representing the range of the computational universality. That interval is divided into m semi-intervals, and the k-th semi-interval is represented by $\left[\frac{k-1}{m},\;\frac{k}{m}\right].$ For the k-th interval, the discretized computational efficiency is defined by

(19) $$E_{\mathrm{MIN}}\left(k\right)=\;\min\{E\left(r\right)\;|\;U_{N}\left(r\right)\;\in {\;\mathrm{Int}}_{k}\},\;\;\mathrm{if}\;U_{N}\left(r\right){\;\mathrm{exists}\;\mathrm{in}\;\mathrm{Int}}_{k};\;\;\min\text{\{}E\left(r\right)|U_{N}\left(r\right)\in {\;\mathrm{Int}}_{j},j>k\},\;\mathrm{otherwise}.\;$$

**Figure 7:**
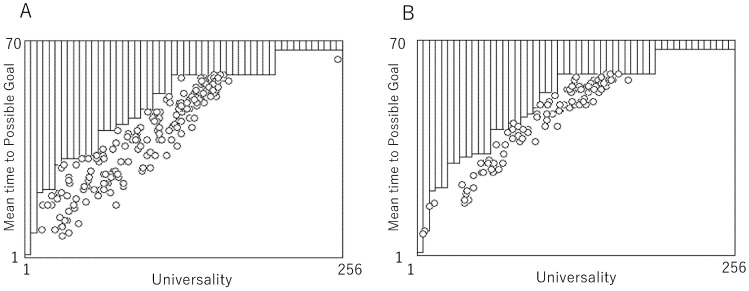
Comparison between computability of AT-ECA and synchronous ECA. Each circle represents the mean time to the possible goal (reachable state) plotted against universality. (**A**): Computability of AT_ECA. (**B**): Computability of synchronous ECA.

Figure 7 shows the computational efficiency, E(r), plotted against the computational universality, U(r), for synchronous ECA, AT_ECA, and asynchronous ECA. The E(r) plotted against U(r) for synchronous ECA is expressed as a function defined by Equation (19). Since all semi-intervals do not contain the point E(r) plotted against U(r) and if the semi-interval has no point then $E_{\mathrm{MIN}}\left(k\right)$ is assigned by the minimum E(r) in larger semi-intervals, $E_{\mathrm{MIN}}\left(k\right)$ is expressed as a step function. Step functions in Figure 7A,B reveal $E_{\mathrm{MIN}}\left(k\right)$ for synchronous ECA. That function implies that the computational efficiency is realized at most in each semi-interval and that the monotonous implies the tradeoff between computational universality and computational efficiency in synchronous ECA.

Circles plotted in Figure 7A show E(r) plotted against U(r) for AT-ECA, where only E(r) that is smaller than the step function $E_{\mathrm{MIN}}\left(k\right)$ for synchronous ECA is represented by a circle. Since plotted circles show more efficient computation rather than synchronous ECA as far as the computational universality is almost the same, we here say for the situation, that the tradeoff of synchronous ECA is broken. Each circle represents AT-ECA whose passive rule is assigned by a rule, *r*. Although there are a possible 256 circles, some circles are hidden in the area upper than $E_{\mathrm{MIN}}\left(k\right).$ It is clear to see that about 70% of all rules break the tradeoff found in synchronous ECA. Figure 7B shows E(r) plotted against U(r) for asynchronous ECA. While asynchronous updating is variously implemented, it is implemented by introducing the probability controlling updating a transition rule such that

(20) $$\;c_{i}^{t+1}=f_{r}\left(c_{i-1}^{t},c_{i}^{t},c_{i+1}^{t}\right)\;\mathrm{with}\;1-p;\;c_{i}^{t},\;\mathrm{with}\;p.$$

Figure 7B is obtained for the condition such that p=0.25. However asynchronous ECA also breaks the tradeoff of synchronous ECA, as fewer rules break the tradeoff compared to AT-ECA.

**Figure 8:**
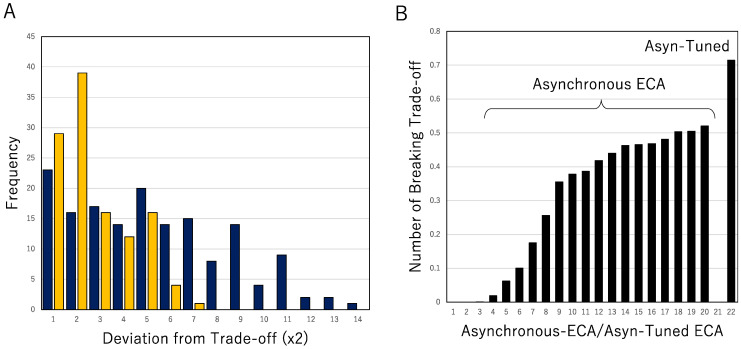
Breaks of the tradeoff in AT-ECA and asynchronous ECA. (**A**): Frequency distribution of the mean deviation from the tradeoff, for AT-ECA (dark blue) and asynchronous ECA (yellow). (**B**): The normalized number of rules breaking the tradeoff for asynchronous ECA and AT-ECA (represented by Asyn-Tuned).

We here compare the break by AT-ECA with that by asynchronous ECA, as shown in Figure 8. Figure 8A shows the frequency distribution for the mean deviation from the tradeoff. For each r, the deviation from the tradeoff is obtained by $E_{\mathrm{MIN}}\left(k\right)-E\left(r\right)$ with $U_{N}\left(r\right)\in {\;\mathrm{Int}}_{k}$, and for each k, the deviations in ${\mathrm{Int}}_{k}$ are averaged. The frequency distribution of the deviation for asynchronous ECA is represented by yellow histograms and that for AT-ECA is represented by dark blue histograms. Asynchronous ECA is estimated for p=0.25 in which more rules break the tradeoff rather than any other parameters. It is clear to see that AT-ECA breaks the tradeoff of synchronous ECA larger than asynchronous ECA with p=0.25. Figure 8B shows the normalized number of rules breaking the tradeoff of synchronous ECA. The left twenty bars represent the normalized number of rule breaking the tradeoff for asynchronous ECA, with the parameter from 0.05 to 1.0 for each 0.05. Bars are arranged in order of size. The largest bar shows about 0.5. The normalized number of rules breaking the tradeoff for AT-ECA is shown at the right end in Figure 8B. Compared to asynchronous ECA, the normalized number for AT-ECA is much larger than that for asynchronous ECA. With respect to both deviation from the tradeoff and the normalized number of rules breaking the tradeoff, AT-ECA breaks the tradeoff much more rather than asynchronous ECA. It implies that AT-ECA has more computability rather than simple asynchronous ECA.

Although it is known that computing based on asynchronous updating is different from that on synchronous updating, they were studied as independently separated. As well as the relationship between thermodynamics and statistical mechanics, the attempt to spell out the relation between them in ECA should be pursued. We here first examine the relationship between asynchronous and synchronous updating with respect to the passive and active updating that is defined order of updating. Synchronous updating is described as a specific updating in the form of passive updating that is assumed to be equal to active updating. Instead of simple equality between passive and active updating, we here implement the traumatic structure of the passive and active, of which both the passive and active updating is accepted (positive antinomy) on one hand, and neither the passive nor active updating is also accepted (negative antinomy) on the other hand. To implement the traumatic structure, perpetual change of the order of updating is required, and that makes each cell update rules both actively and passively (positive antinomy). Simultaneously, the passive and the active are implemented as the form of the local transition rule. Since the passive rule has universality actively dominating all cells and the active rule has passively changed dependent on local configuration, the negative antinomy that is expressed as neither passive nor active is also implemented. In that sense, the traumatic structure is implemented in ECA, and that requires perpetual changing of the order of updating. That is the implementation of the inevitable dissipative structure in ECA. That leads to AT-ECA.

Due to the traumatic structure, each cell uses both passive rule and active rules in tuning the relation between them. It results in time development tuning synchronous and asynchronous updating. Via that tuning, randomness is perpetually supplied to change the order of updating, and that implements inevitable dissipative structure. AT-ECA shows the critical property found in the phase transition from chaos to order. Since applying the asynchronous tuning to ECA leads to the behavior of the edge of chaos, it is easy to implement the edge of chaos. Since either synchronous or asynchronous updating is artificial, tuning between synchronous and asynchronous ECA which looks more natural could show the aspect of natural phenomena. In that sense, the notion of the unfolded edge of chaos plays a significant role in complex systems.

In particular, we estimate the computability of AT-ECA in a term of the quantitative measure of computational universality and computational efficiency. While all ECA systems, synchronous ECA, asynchronous ECA, and AT-ECA show the tradeoff between the computational universality and efficiency, AT-ECA reveals much more efficiency as far as the computational universality of AT-ECA is as same as those of synchronous ECA and asynchronous ECA. It implies that AT-ECA breaks the tradeoff of synchronous and asynchronous ECA. That shows that unfolded edge of chaos generated by AT-ECA carries high performance with respect to computability.

Recently it is well known that the recurrent neural network can be replaced by neural networks containing the reservoir [29]. In particular, there are some attempts in which the reservoir is implemented by cellular automata [30]. Since the reservoir is not a simple thermal pool, the reservoir must play a role in both regulating and disturbing input patterns, such as the edge of chaos. It is expected that a highly efficient and robust reservoir can be realized by AT-ECA.

## 4. Conclusions

We here propose elementary cellular automata taking after the idea of dissipative structure with respect to the updating mechanism. By introducing active and passive updating in a term of the order of updating and non-locality, synchronous updating is defined as an extreme updating of which the passive updating is equivalent to the active one. In contrast, a natural perturbed system is defined by asynchronously tuned elementary cellular automata (AT-ECA) of which both passive and active updating are accepted with respect to the order of updating and neither passive nor active updating is accepted with respect to non-locality. We here call such a paradoxical structure the traumatic structure and claim that the traumatic structure takes after the idea of dissipative structure.

Since AT-ECA shows the critical property of the power law and complex cluster-like patterns featuring both locally periodic and chaotic interaction, it can be said that they mimic the behavior of cellular automata at the edge of chaos. Application of the device of asynchronous tuning to ECA leads to such behaviors, we call the property like the edge of chaos ubiquitously found “unfolded edge of chaos”. In particular, we examine the computational power of AT-ECA with respect to computational universality and computational efficiency. The result shows that AT-ECA or unfolded edge of chaos has much more highly efficient computational power for the same computational universality rather than synchronous and asynchronous ECA.

## Data Availability

Not applicable.
